# Dissemination prevention of antibiotic resistant and facultative pathogenic bacteria by ultrafiltration and ozone treatment at an urban wastewater treatment plant

**DOI:** 10.1038/s41598-019-49263-1

**Published:** 2019-09-06

**Authors:** Norman Hembach, Johannes Alexander, Christian Hiller, Arne Wieland, Thomas Schwartz

**Affiliations:** 1 Karlsruhe Institute of Technology (KIT), Institute of Functional Interfaces (IFG), Microbiology/Molecular biology Department, Hermann-von-Helmholtz Platz 1., 76344 Eggenstein-Leopoldshafen, Germany; 2 Zweckverband Kläranlage Steinhäule, Reinzstrasse 1, 89233 Neu-Ulm, Germany; 3 Xylem Services GmbH, Boschstr. 4 – 14, 32051 Herford, Germany

**Keywords:** Antimicrobial resistance, Environmental impact

## Abstract

Conventional wastewater treatment is not sufficient for the removal of hygienically relevant bacteria and achieves only limited reductions. This study focuses on the reduction efficiencies of two semi-industrial ultrafiltration units operating at a large scale municipal wastewater treatment plant. In total, 7 clinically relevant antibiotic resistance genes, together with 3 taxonomic gene markers targeting specific facultative pathogenic bacteria were analysed via qPCR analyses before and after advanced treatment. In parallel with membrane technologies, an ozone treatment (1 g ozone/g DOC) was performed for comparison of the different reduction efficiencies. Both ultrafiltration units showed increased reduction efficiencies for facultative pathogenic bacteria and antibiotic resistance genes of up to 6 log units, resulting mostly in a strong reduction of the bacterial targets. In comparison, the ozone treatment showed some reduction efficiency, but was less effective compared with ultrafiltration due to low ozone dosages frequently used for micro-pollutant removal at municipal wastewater treatment plants. Additionally, metagenome analyses demonstrated the accumulation of facultative pathogenic bacteria, antibiotic resistance genes, virulence factor genes, and metabolic gene targets in the back flush retentate of the membranes, which opens further questions about retentate fluid material handling at urban wastewater treatment plants.

## Introduction

## Antibiotic Resistance Genes and Facultative Pathogenic Bacteria in Wastewaters

Antibiotic resistance genes (ARGs) and facultative pathogenic bacteria from different origins (hospitals, nursing homes, housing areas, slaughter houses etc.) that pass through treatment plants or agricultural runoffs can lead to a contamination of environmental water resources. In certain circumstances this could cause an impairment of human health in case of direct contact and colonisation^1–4^. As released bacteria are able to persist in the aquatic environment and may proliferate back to critical concentrations, an ecologically worthwhile solution is recommended^5^.

Besides facultative pathogenic bacteria, which can be the causative agents of human and veterinary infections, antibiotic resistance genes are also a point of interest. Their distribution via horizontal gene transfer can occur regardless of their initial origin species, and once released to the environment they are able to transfer to autochthonous bacteria as well as other facultative pathogenic bacteria^6^. This critical ability might lead to an overall increase in antibiotic resistance in their respective habitats. This process is supported by the presence of antibiotics, which even in sub-inhibitory concentrations by medical means, favours horizontal gene transfer e.g. in wastewater treatment plants^7–11^.

Conventional wastewater treatment is not sufficient to entirely remove biological contaminants (bacteria, viruses, and protozoa)^2,12^. Even tertiary treatment with activated charcoal (GAC, PAC) showed insufficient impact on biological contamination in discharges^13^. Up to date ARGs are mentioned in neither drinking water nor wastewater specific regulations or surveillance strategies, despite the World Health Organisation (WHO), as well as the plenum of the United Nations (UN), demanding a disruption of their means of distribution^14^.

Ozonation is already discussed as a possible disinfection process, having already proven its capability to degrade and/or transform micro-pollutants and, in some studies, reduce facultative pathogenic bacteria and ARGs^15–17^. Nevertheless, it is known that ozonation might introduce selection for robust bacteria, including facultative pathogenic bacteria, that have subsequent possible regrowth potential^12^. Another drawback of ozonation is the unpredictable formation of unwanted by-products^5,17,18^.

Ultrafiltration is an alternative process to separate facultative pathogenic bacteria and ARGs from the wastewater effluents. Whereas ozonation targets DNA and other cellular structures, aiming towards inactivation or killing of bacteria^19,20^, ultrafiltration retains bacteria, including antibiotic resistant microbes, by the physical properties of their membrane. This occurs either through direct size exclusion or adsorption processes. Beside bacteria, free floating nucleic acids attached to particles can also be reduced^21^.

This study aimed to develop a deeper understanding of the ultrafiltration reduction efficiencies of facultative pathogenic bacteria and ARGs in comparison to ozonation at an urban wastewater treatment plant (WWTP). Drawbacks of ozone and especially membrane technologies are mentioned and also analysed with the help of modern molecular biology analyses for subsequent risk analysis. More specifically, the often neglected retentate as side product of the ultrafiltration and its inherent health risk is accounted for, as well as the influence of the ultrafiltration fluxes on the elimination effect.

## Material and Methods

### Technical operation

The investigated WWTP is located in the south-west of Germany and treats the clinical, industrial, and municipal wastewater of 440,000 population equivalents. Volumes of 80,000 to 100,000 m^3^ of wastewater are treated per day before the conditioned waters are released into the river Danube. The treatment consists of conventional mechanical and biological stages, along with a precipitation clearance. The mechanical treatments included a sand and grease trap, followed by a fine screen, and finally the primary sedimentation basin. The biological treatment consisted of three treatment steps, starting with biological phosphorous elimination, followed by a two-step nitrification/denitrification stage, and then further clarification through a secondary clarifier.

The two ultrafiltration (UF) units were both designed in semi-industrial sizes and contained hollow fibre modules with an organic membrane material. The UF units were provided by membrane producing companies (MICRODYN-NADIR GmbH, Wiesbaden, Germany and INGE GmbH, Greifenberg, Germany). Both companies are directly cooperating with German and international water industries. Organic membranes were chosen in respect of later up-scaling and economic reasons, as the operation of the alternative ceramic membranes are not economical in large scale systems. Here, the organic membranes allow flux rates up to 90 L/m²h at a transmembrane pressure of up to 1000 mbar^22^.

Ultrafiltration 1 (UF1) had a membrane surface of 80 m² and was operated in inside/out mode and utilised a membrane with a nominal pore size of 20 nm. The filtration was performed at a flux of 70 L/m²h. Ultrafiltration 2 (UF2) was operated in an outside/in mode with a nominal pore size of 25 nm and with the addition of a precipitating agent (polyaluminumchloride) performed at a Flux of 35 L/m²h. Flushing of the retentate of both membranes was performed every 45 minutes with water and air. Additional backwashing to remove scaling of both membranes was performed with a mixture of 30% NaOH and 5% NaClO, followed by a second washing step with 30% HCl were executed less frequently and on demand to ensure stable conditions. During the project, both ultrafiltration units were operated with the outflow of the upstream biological treatment.

The ozone facility (OCS-GSO30, WEDECO, Herford, Germany) operated with an ozone concentration of 1 g ozone/g dissolved organic carbon (DOC) and a flow rate of 7 m³/h, which resulted in an ozone contact time of 5 minutes. Ozone concentration of 1 g/g DOC was chosen in respect to micro-pollutant removal. As considerable higher ozone concentrations at 1.5 g ozone/g DOC were observed to have an eco-toxicological effect and would therefore not be appropriate regarding the complete wastewater treatment process without further treatment steps^23^. The facility utilized diffusers for ozone intake and a catalytic ozone destructor to withhold the remaining gaseous ozone. DOC measurements were performed just before each sampling.

During the sampling period from February till November the treatment plant operated without failure and the relevant parameters of the wastewater utilized for advanced treatment techniques were recorded hourly. The mean pH value of the wastewater was 8.1 ± 0.7 and the average water temperature was 16.0 °C ± 3.2 °C. Further monitored water specific parameters were DOC with 4.3 g ± 1.2 g, conductivity with 828.1 µS/cm ± 193.1 µS/cm and the SAC (spectral adsorption coefficient) with 9.4 ± 1.7. These circumstances have to be considered as especially ozone treatment is dependent on the DOC, temperature, and organic compounds. Most favourable conditions for an ozone treatment would be, low DOC and SAC values, temperatures above 25 °C and a pH-value of 7.0)^24^. As the temperature of the system is highly dependent on the season, the pH-value is nearly optimal for an ozonation. The elimination efficiency of ultrafiltration itself is almost independent of the composition of the treated water. Solely, frequent occurring scaling and fouling causes a decrease of membrane overall lifespan and increases the energy required to maintain a specific flux rate and have to be approached accordingly^25^.

**Figure 1:**
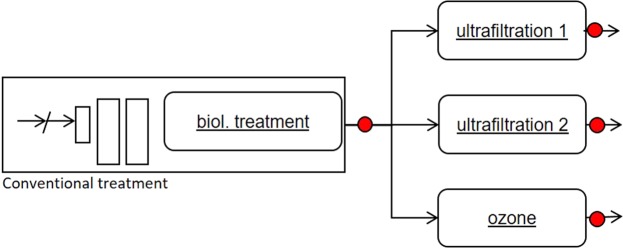
Location of each treatment process. Sampling points are marked as red dots.

Both ultrafiltration and the ozone treatment were studied simultaneously for better comparison of the technologies and reduction efficiency results (Fig. 1).

### Sampling, sample preparation, and DNA extraction

For evaluation of the ultrafiltration and ozone reduction efficiency, 4 sampling campaigns were performed during 9 months (February till November, 2017). Additionally, two samplings with different flux rates (12.5, 50, 90 L/m²h) were performed for ultrafiltration. Inflow and outflow of the membrane facilities, as well as back flushed water, containing the retentates of both ultrafiltration units were analysed in parallel. Volumes of 10 L of each UF filtrate, 750 mL of ozoned water, 300 mL of inflow, and 400 mL of flush-water were vacuum-filtrated through a 0.2 µm polycarbonate membrane (Whatman Nuclepore Track-Etched Membranes, Sigma-Aldrich, Munich, Germany). After the filtration process, the membranes were treated with 0.25 mM propidium monoazide (BLU-V viability PMA-kit, Quiagen GmbH, Hilden, Germany) to complex free DNA as well as DNA from dead or injured bacteria^26^. DNA extraction from the filtered biomass at the membrane was performed using the Fast DNA^TM^ Spin Kit for Soil and FASTPREP^®^ Instrument (MP Biomedicals, Santa Ana, CA) according to previous studies and stored until further analysis at −20 °C^2^.

### Taxonomic marker genes and antibiotic resistance genes

All qPCR systems targeting ARGs and marker genes specific for facultative pathogenic bacteria were analysed for specificity and sensitivity using reference bacteria according to Hembach *et al*.^2^. Full primer sequences are listed in the Supplementary Information (SI Table 1). *Escherichia coli* (yccT gene) (4), *Acinetobacter baumannii* (*secE* gene^27^), intestinal enterococci (23S rRNA gene) (4), and the 16S rRNA gene for eubacterial determination were quantified. The antibiotic resistance genes *sul1* (sulfamethoxazole resistance)^28^, *bla*TEM (β-lactam resistance)^28^, and *tetM* (tetracycline resistance)^29^ were studied as they represent the most frequently detected ARGs in urban wastewaters^2^. The antibiotic resistance genes *CTX*-*M*^30^, *CTX*-*M*-*32*^28^, *bla*OXA-*48* (carbapeneme resistances)^31^, and *bla*VIM (Imipenem resistance)^32^ were also analysed. Furthermore, the resistance genes *CMY*-*2* (against Cephalosporines)^33^, *vanA* (Vancomycine resistance)^34^, *mcr*-*1* (Colistin resistance)^2^, and *bla*NDM (New Deli-beta-lactamase)^31^ were monitored as they represent resistances against last line of defence antibiotics.

For qPCR analysis a Bio-Rad Cycler CFX96 (CFX96 Touch™ Deep Well Real-Time PCR Detection System, Bio-Rad, Munich, Germany) was used. All samples were measured in technical triplicates. The specific taxonomic and resistance directed genes in the entire bacterial community were quantified utilizing a SYBR Green qPCR approach according to Hembach *et al*. and Jäger *et al*.^2,3^. Reactions were run in volumes of 20 µL, containing 10 µL Maxima SYBR Green/ROX qPCR Master Mix (2×) (Thermo Scientific, Nidderau, Germany), 8.2 µL of nuclease-free water (Ambion, Life technologies, Karlsbad, Germany), 0.4 µL of the respective primers (stock concentration 10 µM), and 1 µL of template DNA. The qPCR protocol comprised 10 minutes at 95 °C for activation of the DNA polymerase followed by 40 cycles of 15 s at 95 °C, and 1 min at 60 °C for primer annealing and elongation. Melting curves were recorded by raising the temperature from 60 °C to 95 °C (1 °C every 10 seconds) to determine the specificity of the amplification. This allows the evaluation of the amplicons based on their lengths and base pair ratios. Hence, higher AT content results in higher melting points at constant amplicon lengths. Data analysis was performed by using the Bio-Rad CFX Manager software (version 3.1 © 2012 Bio-Rad Laboratories).

An absolute quantification normalized to 100 mL water sample was performed to demonstrate the reduction effects of the applied treatment processes for quantitative analysis. Cell equivalent calculations based on the qPCR based Ct-values in the linear part of calibration curves^2^. The respective calibration equations of each target are listed in the SI Table 1. Calibration curves were obtained with reference strains carrying the corresponding specific ARGs or taxonomical marker genes.

These reference strains with known genome sizes, respective known plasmid sizes, were used to calculate cell equivalents utilizing the amount of DNA used for creating the target specific calibration curve. Equation 1 was used to create a correlation of the amount of DNA in the calibration solutions and the corresponding cell equivalents. It utilized an average molecular weight for one base pair of about 650 g/mol, Avogadro’s number with 6.022 × 10^23^ molecules/mol, and a converting factor of 10^9^ ng/g.

$$cell\,equivalents=\frac{(amount\,of\,DNA\,[ng])\ast 6.022\ast {10}^{23}}{(average\,size\,of\,genome[bp])\ast {10}^{9}\ast 650}$$

**Equation 1**. Calculation of cell equivalents in a PCR-reaction based on the average molecular weight of one base pair, Avogadro’s number, the amount of DNA used for the reaction and a converting factor.

Limit of detection (LOD) of each detection system, ranging from 4 gene copies (*E*. *coli yccT* gene) to 126 gene copies (*intl1* gene), are listed in the Supplemental Information (SI Table 2).

### Metagenome analyses

Extracted genomic DNA from the retentate of both ultrafiltrations was sequenced by Eurofins GATC Biotech GmbH, Munich, Germany and mapped against the MvirDB microbial virulence database and the integrated reference catalogue (IGC)^35–37^. Taxonomic profiling was done using Kraken and Mikrokraken reference database^38^.

The sequencing generated 22,545,798 reads for the retentate of UF1 and 23,480,814 reads for UF2 of which 3,387 (0.02%) and 3,428 (0.01%) reads were successfully mapped to a reference in the MvirDB database for resistance screening. 173,478 reads (0.77%) of UF1 and 153,226 reads (0.65%) of UF2 were successfully mapped against the integrated reference IGC catalogue for functional profiling. Taxonomic profiling yielded 739,418 (3.28%) classified reads for UF1 and 596,486 (2.52%) for UF2.

The raw sequencing data can be obtained as .fastq file at the NCBI database with the BioProject Accession number: PRJNA524456.

### Cultivation

All cultivation analyses were performed immediately after sampling to minimize preselection bias during transport and storage. Different selective agar systems were used for the detection of *E*. *coli* (Chromocult-Coliform-Agar, Merck, Darmstadt), *Acinetobacter* spp. (Acinetobacter-CHROMagar, MAST Diagnostica GmbH, Reinfeld, Germany), intestinal enterococci (Slanetz-Barley Oxoid, VWR, Darmstadt and Bile esculin Agar, VWR, Darmstadt), and extended spectrum beta lactamase (ESBL) producing Gram-negative bacteria (CHROMagar^TM^ MAST Diagnostica GmbH, Reinfeld). Three different sample volumes (750 mL, 1000 mL, and 1250 mL) were filtered through a 0.45 µm nitrocellulose membrane (GE Healthcare Life sciences, Solingen, Germany). The membrane was then transferred onto an ager plate and incubated under conditions recommended by the manufacturer. After an appropriate incubation time, Colony Forming Units (CFU) were determined for each plate and calculated as CFU per 100 mL. The mean of all three sample volumes were calculated for final evaluation.

### Statistics

DNA extraction was performed in technical triplicates and extracts were pooled afterwards for further measurements. qPCR was also performed in technical triplicates and their mean Ct-values were used for cell equivalent calculation of each target and sample. For statistical evaluation, the cell equivalent median from all four sampling campaigns was utilized for p-value calculation to verify the significance displayed in the figures. Due to the sampling periods (n = 4), significance was tested with a one tailed non-parametric Mann- Whitney- U test^29^.

Gene abundances of facultative pathogenic bacteria as well as ARGs are displayed as boxplot. Herein the edges between two different bars represent the median. The bars themselves represent the upper (p = 0.75) and the lower (p = 0.25) quantiles. Error bars illustrate the maximal and minimal abundances of the sampling point over the complete sampling campaign.

## Results and Discussion

### Comparative analyses of treated wastewater

Conventional treatment at the WWTP consisted of the mechanical removal of solids and biological treatment with sedimentation of sludge biomass. Hence, the treated wastewater, after biological treatment, was used as a reference value to compare the reduction efficiencies of the advanced treatment processes for facultative pathogenic bacteria and ARGs.

**Figure 2:**
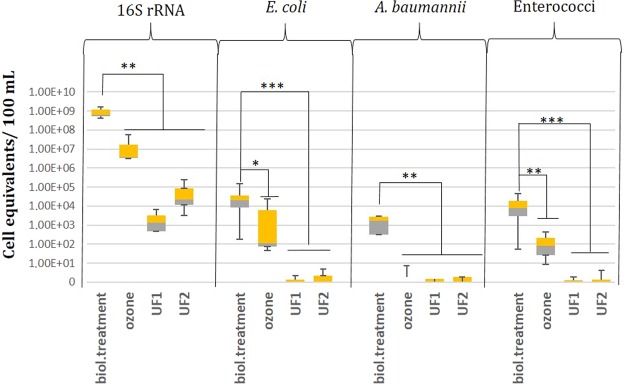
Cell equivalents of facultative pathogenic bacteria after the treatment with ozone (1 g/g DOC) and both UF- units. Significance values are represented by an asterisk.

The eubacterial 16S rRNA gene marker was present at a concentration of 6.28 × 10^8^ cell equivalents/100 mL after conventional treatment without any implementation of advanced treatment technologies (Fig. 2). Based on this, around 5.65 × 10^17^ cell equivalents of *Eubacteria* will reach the receiving river each day (based on 90,000 m³ per day). Specific gene markers targeting *E*. *coli*, *A*. *baumannii*, and enterococci were found in high abundances after conventional treatment as illustrated in Fig. 2. Here, these facultative pathogenic bacteria were present in the range of 1.63 × 10^3^ to 2.01 × 10^4^ cell equivalents/100 mL. The total abundances of the three facultative pathogenic bacteria (*E*. *coli*, *A*. *baumannii*, enterococci) reached together a calculated number of 2.66 × 10^13^ cell equivalents per day that are released to the riverside, when 90,000 m^3^ of conditioned wastewater per day was released from the WWTP into the receiving river.

**Figure 3:**
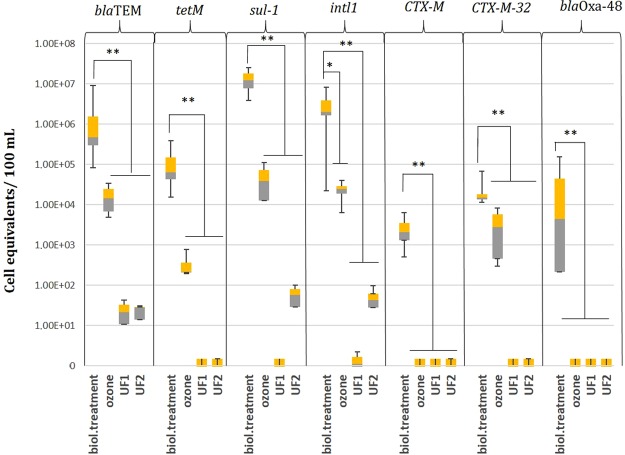
Cell equivalents of ARGs after the treatment with Ozone (1 g/g DOC) and both UF- units. Significance values are represented by an asterisk.

Regarding the median of each specific target gene, ARGs in the effluent of the conventional wastewater treatment can be grouped in three different subgroups according to their remaining concentrations. Antibiotic resistance genes and the integron *intl1* gene being quantified in high amounts of cell equivalents/ 100 mL were grouped as frequently detected genes. Herein, the genes *bla*TEM (β- lactam resistance against ampicillin) with 4.72 × 10^5^ cell equivalents/100 mL, *tetM* (tetracycline resistance) with 6.31 × 10^4^ cell equivalents/100 mL, *sul1* (*sulfamethoxazole* resistance) with 1.25 × 10^7^ cell equivalents/100 mL, *ermB* (*erythromycin* resistance) with 9.66 × 10^4^ cell equivalents/100 mL and *intl1* (class 1 integron specific marker) were categorized as frequently occurring ARGs (Table 1, Fig. 3). Less frequently occurring ARGs made up the subgroup of intermediately detected genes. This group consists of the genes *CTX*-*M*-*32* (1.52 × 10^4^ cell equivalents/100 mL), *CTX*-*M* (1.61 × 10^3^ cell equivalents/100 mL), *bla*OXA-*48* (1.67 × 10^3^ cell equivalents/100 mL), *CMY*-*2* (4.98 × 10^3^ cell equivalents/100 mL), and *bla*VIM (4.92 × 10^3^ cell equivalents/100 mL). The third subgroup consists of antibiotic resistance genes which were rarely detected or only were present in low concentrations in the effluent of the conventionally treated wastewater. *vanA* (9.14 × 10^−1^ cell equivalents/100 mL), and both *mcr*-*1* and *bla*NDM belong to this category of rarely detected antibiotic resistance genes (Table 1).

**Table 1:** Categorization of different antibiotic resistance genes based on their median abundance in cell equivalents/100 mL in the investigated WWTP.

	Resistance gene	Median of cell equivalents in 100 mL	Median absolute deviation (MAD)
Frequently detected	*sul1*	1.25E + 07	8.54E + 06
	*intl1*	2.00E + 06	1.92E + 06
	*bla*TEM	4.72E + 05	3.89E + 05
	*ermB*	9.66E + 04	9.57E + 04
	*tetM*	6.31E + 04	4.74E + 04
Intermediately detected	*CTX*-*M*-*32*	1.52E + 04	3.08E + 03
	*bla*OXA- *48*	1.67E + 03	1.65E + 03
	*CTX*-*M*	1.61E + 03	1.10E + 03
	*bla*VIM	4.92E + 02	4.25E + 03
	*CMY*-*2*	4.98E + 03	4.61E + 02
Rarely detected	*vanA*	9.14E-01	9.14E-01
	*mcr*-*1*	<LOD	<LOD
	*blaNDM*	<LOD	<LOD

Median absolute deviation (MAD) displays the statistical dispersion based on the median values according to MAD = median (|x_i_ − median(x_i_)|).

Specific antibiotic resistance genes that have higher clinical relevance (like against vancomycin via the *vanA* gene and colistin via the *mcr*-*1* gene) harbour a superior threat to human health than, for example, resistance against sulfamethoxazole (*sul1*). These critical ARGs are comparatively rare (Table 1, rarely detected), but can still be detected in the effluent after conventional treatment^2,39^. Due to their low concentrations, rarely detected, as well as some intermediately detected, ARGs are not suited for an evaluation of advanced treatment techniques, as they get frequently reduced below their detection limits without any evaluation of reduction efficiency. The ARGs with higher abundances are better suited for that purpose as, even after higher reduction efficiencies, the remaining cell equivalents can be detected^5,40^. Thus, for evaluation the frequently and intermediate occurring resistance genes *bla*TEM, *tetM*, *sul1*, *CTX*-*M*, *CTX*-*M*-*32*, *bla*OXA-*48*, and the class 1 integron gene *intl1* were considered. As these ARGs, were originally also from anthropogenic fields, and are spread in the sewage system, they allow to predict the fate of rarely occurring resistance genes becoming distributed to the environment if no actions are taken to contain their spread^41^.

The investigated advanced technologies were two ultrafiltration membrane facilities and one ozone facility, which were run in parallel at the WWTP to assure analyses of the same inflow wastewater of the mentioned facilities (Fig. 1).

All advanced processes showed a strong reduction of facultative pathogenic bacteria compared to the cell equivalent abundances after conventional treatment. *E*. *coli*, *A*. *baumannii*, and enterococci were reduced below the detection limit by both ultrafiltration units (UF1 and UF2). The detection of the 16S rRNA gene marker representing *Eubacteria* was detected in higher abundances and was quantified with 1.35 × 10^3^ for UF1 or 2.19 × 10^4^ cell equivalents/100 mL for UF2 in the outflow water samples. This observation leads to the conclusion that ultrafiltration was not able to remove the entire bacterial community from the wastewater stream. Small bacteria or spores that pass the membrane, or flawed membrane installation, may allow bacteria to bypass the actual membrane and can be considered as a possible eubacterial contamination source^42^.

Ozone treatment with 1 g ozone/g DOC strongly reduced only *A*. *baumannii* with 3 orders of magnitude, while *E*. *coli* and enterococci got reduced with 2 orders of magnitudes at maximum. This reduction efficiency was less sufficient in comparison to the cell equivalents/100 mL found after ultrafiltration, with reductions up to 5 orders of magnitude. This also included the eubacterial 16S rRNA gene targets.

Ozone treatment using low concentration being optimized for micro-pollutant removal achieved a reduction of about 2 log units for the frequently occurring ARGs, resulting in concentrations varying from 2.18 × 10^2^ (*tetM*) to 3.92 × 10^4^ (*sul1*) cell equivalents/100 mL in the ozone facility outflow (Fig. 3). Both UFs strongly reduced these ARG targets to concentrations of less than 100 cell equivalents/100 mL (i.e. up to 6–7 log unit reductions). UF2 was not capable to remove the gene *sul1* and the *intl1* gene target below the detection limit, unlike the UF1 treatment. A number of 570 and 430 cell equivalents, respectively, of these frequently occurring genes were still present in 100 mL UF2 outflow, but still a 5 log unit reduction was found.

Considering the intermediately occurring ARGs (Table 1), ozonation resulted in a 4 log unit reduction of the β-lactamase genes *CTX*-*M* (against cephalosporins) and *bla*OXA-*48* (against carbapenems). Bacteria carrying the resistance gene *CTX*-*M*-*32* (β-lactamase against cephalosporins) were able to pass the ozonation facility to a greater extent, and was found in concentrations of up to 2.77 × 10^3^ cell equivalents/100 mL in the outflow of the ozone facility. It has previously been reported that *P*. *aeruginosa* and GC-rich bacteria are capable of coping with the bactericidal effects of ozone and, as a consequence, can be present in the outflow of ozone facilities Therefore GC-rich bacteria carrying ARG like the *CTX*-*M*-*32* gene might be able to pass the ozonation stage, demonstrating one mechanism of how ARGs can bypass this oxidative treatment method. These resistances can further be transferred to a wider variety of bacteria. But, UF1 and UF2 managed to reduce all intermediate occurring ARGs below their detection limits, thus supposing eliminating them from the conventionally treated wastewater (Fig. 3).

In addition, the level of significances varied between conventional treatment and ultrafiltration or ozonation. As *E*. *coli* and enterococci results generated p-values of 0.1 and 0.05 for ozone treatment, the treatment with UF membrane technology yielded in p-values of 0.01 for both *E*. *coli* and enterococci. The eubacterial 16S rRNA and *A*. *baumannii* reduction demonstrated a significance level of 0.05, which was independent of the applied treatment techniques (Fig. 2). Hence, ultrafiltration possessed stronger and more constant bacterial removal efficiencies. Ultrafiltration reduced facultative pathogenic bacteria and ARGs until a certain concentration threshold in the effluent was reached, whilst ozonation reduced contaminants only for a specific amount. More specifically, ultrafiltration was capable of reducing facultative pathogenic bacteria, as well as ARGs, close to the detection limit, independent of their initial concentration.

More generally, under the given process parameters, both advanced treatment technologies (ozonation and ultrafiltration) were able to reduce facultative pathogenic bacteria, as well as bacteria carrying antibiotic resistance genes, significantly. The membrane technologies especially were able to reduce a number of gene targets below or close to the detection limits. It was statistically confirmed that UF showed a more consistent ability to reduce gene targets in this way compared to ozonation, resulting in the higher p-values. It has to be mentioned that the applied ozone facility at the WWTP was adjusted to parameters be relevant for micro-pollutant removal^13^. Optimization of the ozone facility parameters like ozone concentration or hydraulic retention times for a more effective removal of both micropollutant but also microbial contaminations have to be discussed in future research approaches. Optimization efforts have recently been performed by Iakovides *et al*. utilizing rather low ozone concentrations between 0.125 and 0.75 g ozone/g DOC^43^. These authors showed that already low dosages and short hydraulic retention times are sufficient to eliminate parent compounds of e.g. antibiotics, but higher concentrations were needed to secure a sufficient and lasting reduction of bacteria and resistance genes. In general, higher ozone concentration inherits a rising risk of the formation of unwanted by products. Therefore special interest of a joint optimization process development needs to focus on prevention of formation and release of unwanted or harmful substances to the environment^44,45^. Unfortunately, this transformation processes can result in harmful by-products, which by themselves can have eco-toxicological effects up to a degree were the used ozonation as standalone process is questionable^46^. But, additional adsorption steps after ozonation would also be helpful to remove remaining ozone molecules and unwanted by-products, especially at higher ozone concentrations, which are supposed to harm the structural integrity of membranes in subsequent processes. Nevertheless, previous studies using additional filtration steps with charcoal or sand filtration downstream the ozonation inherit the risk of bacterial regrowth^13^.

For two additional sampling campaigns, the flux rates of both UF units were changed from 70 L/m²h to 12.5, 50, and 90 L/m²h for UF1, as well as 12.5 and 50 L/m²h for UF2. With only two independent samples, a deeper statistical analysis of this parameter variation was not feasible. On one hand, the UF1 membrane facility showed increased reduction efficiencies at higher flux rates. UF2, on the other hand, lacks the increased reduction effect with rising flux rates (see. SI Table 3). As both membranes showed different behaviours with an increasing flux rate, the membrane parameters seem to possess a stronger impact on the purification effects than the applied flux rates. For economic reasons, an increased flux rate could be advantageous depending on the increased energy consumption, as higher volumes could be processed by one filtration unit. Studies of ultrafiltration units used for drinking water treatment also propose the adjustment of operating parameters to maintain a constant transmembrane pressure at 1 bar or below, as it helps to reduce irreversible fouling^47^.

### Cultivation of facultative pathogenic bacteria

**Figure 4:**
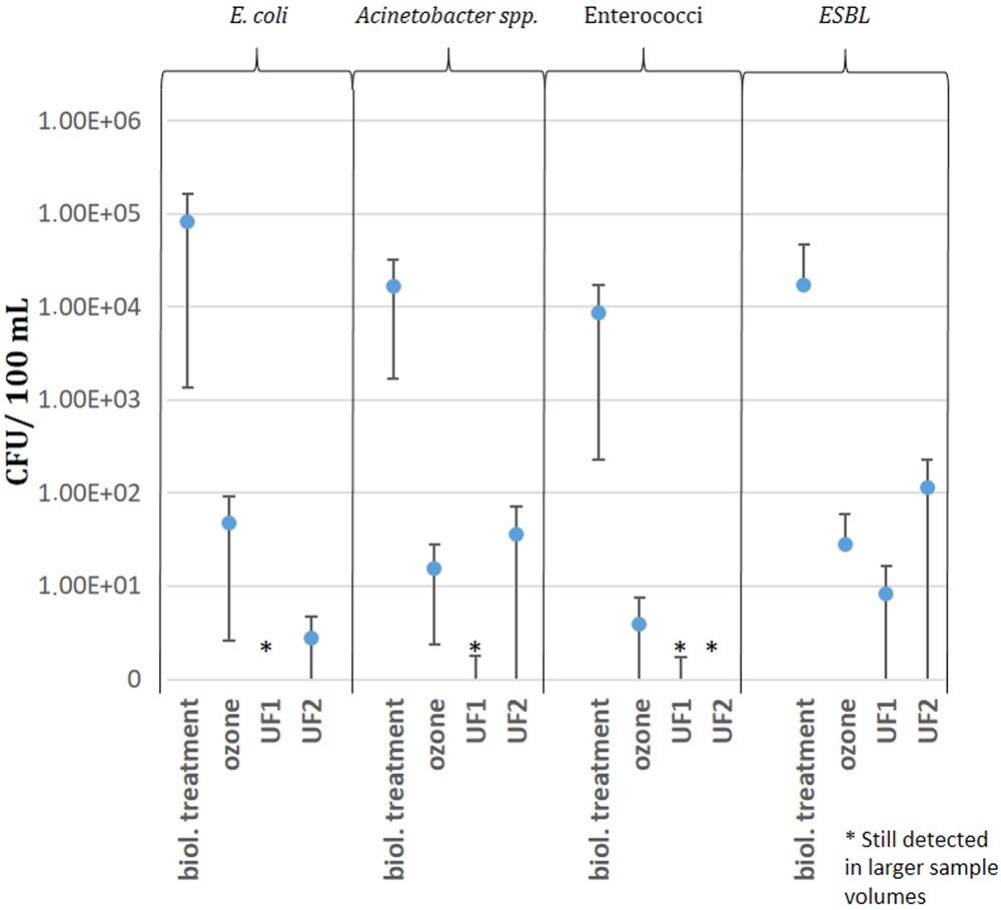
Colony forming units (CFU) in 100 mL and their standard deviation growing on different selective agar plates. All samples where CFUs were detected in concentration below 1 CFU/100 mL but in higher sample volumes are marked with an asterisks.

Cultivation-based experiments were performed to demonstrate the presence of cultivable facultative pathogenic bacteria and bacteria resistant against ESBL antibiotics. Figure 4 shows the Colony Forming Units (CFU) calculated for 100 mL water sample volumes. Plate counts of the conventional treatment ranged from 8.70 × 10^3^ CFUs/ 100 mL for enterococci, 1.70 × 10^4^ CFUs/ 100 mL for *Acinetobacter* spp., and 8.19 × 10^4^ CFUs/ 100 mL for *E*. *coli*.

Ozone treatment reduced cultivable *E*. *coli*, *Acinetobacter* spp., and enterococci by about 3 log units independent of their initial concentration after biological treatment. UF1 reduced facultative pathogenic bacteria below 1 CFU/100 mL water. Nevertheless, it was still possible to detect 3–10 CFUs in larger sample volumes of 1250 mL (data not shown).

Cultivated clinically relevant antibiotic resistant bacteria displayed in Fig. 4 show that resistant bacteria were present in the outflow of the conventional treatment with 1.74 × 10^4^ CFU/100 mL of ESBL bacteria. All advanced technologies were able to strongly reduce the CFU numbers. Still some ESBL bacteria were also detected in the filtrate of UF1 and UF2, as well as after ozonation (28 CFUs after ozonation, 8 CFU after UF1, and 115 CFU per 100 mL after UF2).

The comparable reduction effect of ozonation and ultrafiltration in cultivation experiments might be impacted by the known limitations of cultivation methods. It is known that ozone induces a viable but nonculturable (VBNC) state rather than disrupt the bacterial membrane leading to cell lysis^48^. Such VBNC states can’t be detected through cultivation-based methods. Therefore, ozonation might yield higher reductions using cultivation methods rather than with molecular methods. Such molecular methods based on the same samples detected 113 *E*. *coli* cell equivalents/ 100 mL and 81 cell equivalents/ 100 mL of enterococci, whilst cultivation based methods only detected 48 CFU of *E*. *coli* and 4 CFUs of enterococci/ 100 mL. This leads to the assumption that the clinically relevant ESBL were also partly present in their VBNC states, and would regain their activity after longer incubation periods. This discrepancy could potentially lead to a higher human health risk if not taken into account.

### Analysis of retentate

The presence of living bacteria in the retentate of membrane filtration is a major drawback of ultrafiltration technology. This is contrary to oxidative technologies like ozonation, where high fractions of bacteria become inactivated^13^. Bacteria, including facultative pathogenic bacteria and antibiotic resistant contaminants, are retained during ultrafiltration and get removed from the membrane during back-flushing steps. Thus, two sampling campaigns were performed where the inflow, as well as the retentate after a full filtration cycle (60 min), was analysed to control this situation.

It was demonstrated by the qPCR quantification of gene targets that the retentate water of UF1 harbours 2 log unit higher concentrations of facultative pathogenic bacteria compared to the inflow water. The 16S rRNA eubacterial gene marker increased from 3.46 × 10^6^ cell equivalents in the UF inflow to 2.53 × 10^8^ cell equivalents/ 100 mL in the retentate of UF1, indicating the general concentration of the bacteria. In contrast to the other facultative pathogenic bacteria, *A*. *baumannii* increased by only one order of magnitude in both UF units from 3.09 × 10^1^ to 3.03 × 10^2^ cell equivalents/100 mL. The abundance of ARGs under investigation in UF1 also demonstrated increased concentration values in ranges of 2 log units within the retentates. The gene *bla*TEM increased from 3.09 × 10^3^ to 1.20 × 10^5^ cell equivalents/100 mL, *tetM* accumulated from 5.34 × 10^2^ to 2.76 × 10^4^ cell equivalents/100 mL, and *bla*OXA-*48* from 6.56 × 10^1^ to 2.95 × 10^3^ cell equivalents/100 mL. For cultivation experiments the above mentioned media systems and dilution steps were used to demonstrate cultivable facultative pathogenic bacteria in the retentates. Despite dilution at three different concentrations of the retentate the agar plates were mostly overgrown and not qualified for quantitative analysis. Nevertheless, living *E*. *coli*, *enterococci*. and *Acinetobacter spp*. were highly abundant in retentates.

In contrast to UF1, the concentration of facultative pathogenic bacteria, as well as most ARGs, in the retentate of UF2 were found in a lower degree of accumulation. Abundances of *E*. coli increased from 1.58 × 10^4^ cell equivalents in 100 mL of the influent to 1.39 × 10^5^ cell equivalents in 100 mL retentate. This observation hints at the suggestion that the membrane efficiency of UF 2 harbours an inferior retention capacity in comparison to UF 1.

A full list of the concentrations for each of the facultative pathogenic bacteria and ARGs in the retentate is listed in SI Table 4.

In addition to qPCR studies, metagenomic sequencing was performed to gain a more comprehensive overview about the genetic pool (resistome, virulence factors, and metabolic genes) to predict a possible risk of operation with the retentate. It is important to generate a deeper understanding of the overall antibiotic resistance and virulence gene levels of the retentate waters for their safe subsequent handling at the WWTP. It is calculated by the local WWTP engineers that retentate volumes of about 5,000 m^3^/day are expected after ultrafiltration of the daily waste waters. Therefore, decisions must be made about a possible recycling of the retentate back to activated sludge tanks, or inactivation of the retentate microbes by other means.

**Figure 5:**
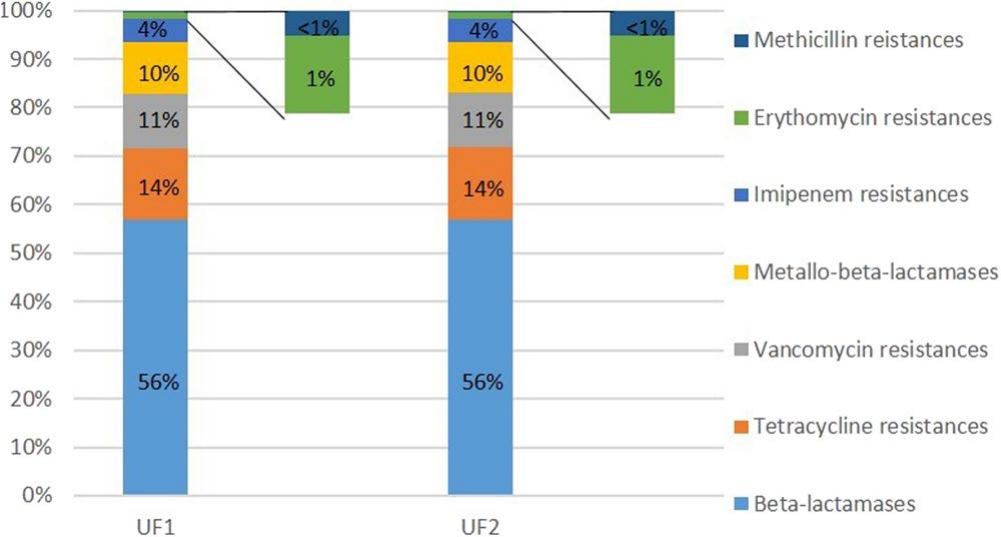
Percentage of mapped resistance genes in the metagenomes of the retentates from both ultrafiltration units (UF1 and UF2). A summary of the detected genes is listed in the SI Table 5.

About 1,205 reads (UF1) and 1,201 reads (UF2) of retentate sequencing were successfully mapped against an antibiotic resistance gene data bank (Mvir DB). Herein, a cluster of ß-lactamases was found to contribute the majority (685 identified genes), followed by different tetracycline resistance genes (178 genes). Importantly, genes against the reserve antibiotics vancomycin (135 genes) and metallo-β-lactamase, i.e. with KPC carbapenemases and *bla*NDM genes (128 genes), were identified. Both KPC and *bla*NDM genes were handled separated from the remaining β-lactamases, as both provide resistances to nearly all known β-lactam antibiotic drugs^49^. Methicillin, erythromycin, and imipenem resistance genes were also detected, but in lower frequencies. Figure 5 displays the relative occurrence of the mapped antibiotic resistances. Due to the same inflow of both UF systems, the composition of the retentate resistome composition did not differ in any extent. The ß-lactamases contributed 56% of the overall resistome. Resistance genes against the last resort antibiotic vancomycin made up about 11% of the resistance genes, and was not expected to be so frequently identified. At least 31 vancomycin resistance genes of this operon were identified, including the *vanA* gene. Resistances against the veterinary and human-relevant tetracycline antibiotic comprised about 14% of sequences, the resistance genes against the less commonly used erythromycin is documented with 1%, and methicillin resistance genes were identified in less than 1% of reads. The rarely found methicillin resistances were identified via the resistance genes *mecA* and *mecR1*. Although imipenem is used quite rarely, a total of 13 different *bla*VIM resistance genes could be detected in the retentates. A list of identified resistance genes is shown in SI Table 5. The low relative abundances of erythromycin resistance genes in the metagenome of the retentate derive from the high variety of gene variants of other ARGs. For example, vancomycin resistance is mediated by 36 different genes, which were detected via metagenomic analysis, therefore despite *vanA* being categorized as a rarely detected resistance gene, vancomycin resistance take up 11% of the resistome. Erythromycin resistance was also found in the sample, but is only mediated by 7 different genes (SI Table 5). Thus, the frequently detected *ermB* genes, together with 6 other gene variants, take up a significantly lower proportion of the resistome.

According to the Mvir database mapping, the retentates of both UFs revealed a total of 792 (UF1) and 750 (UF2) pathogenicity islands as well as 120 (UF1) and 306 (UF2) different genes coding for virulence proteins. Amongst the 25 most identified virulence factors in both retentates were various transposases of the facultative pathogenic bacteria *E*. *coli*, *K*. *aerogenes*, *P*. *aeruginosa*, and *Shigella flexneri* as well as the *Pseudomonas spp*.-associated multiresistant ß-lactam transposon Tn1412. Additionally, 74 genes coding for protein toxins were found in the retentate of UF1 and 136 genes coding for protein toxins in the UF2 retentate.

Functional gene profiling revealed that, besides carbohydrate metabolism, a wide variety of genes involved in metabolic and biotransformation activities remained present in the retentate. The metabolic activity genes (6.38% for UF1 and 5.78% for UF2) and repair mechanisms genes (4.29% for UF1 and 4.35% for UF2) give hints towards the population activity and their capabilities of handling with stress responsive actions, such as their response to antibiotics or oxidative stress responses. A variety of at least 19 recombinase and integrase associated genes as well as 417 transposases associated genes amongst the functional genes further support the presumption, that the retentate can actively participate on horizontal gene transfer. Table 2 shows the most abundant functional gene categories.

**Table 2:** Percentage of the most frequently identified functional gene categories of the retentate of both ultrafiltration units and their corresponding percentages. Data acquired after metagenomic sequencing utilising IGC as reference dataset. Total mapped reads UF1: 173,478 UF2: 153,226.

Function	UF1	UF2
Carbohydrate metabolism	8.86	8.87
Cellular process and signalling	6.39	6.72
Metabolism	6.38	5.78
Membrane transport	5.62	5.29
Genetic information processing	5.17	4.79
Amino acid metabolism	4.49	4.45
Replication and repair	4.29	4.35
Nucleotide metabolism	2.96	2.92
Enzyme families	2.63	2.61
Energy metabolism	2.46	2.33
Transcription	2.02	1.75
Translation	1.88	1.88
Metabolism of cofactors and vitamins	1.75	1.83
Folding, sorting, and degradation	1.66	1.82
Glycan biosynthesis and metabolism	1.43	1.52
Signal transduction	1.39	1.25
Lipid metabolism	1.08	1.11
Metabolism of terpenoids and polyketides	0.50	0.57

Bacteria inherent to treatment plants, like *Nitrosomonas* spp. and *Accumulibacter* spp., that are involved in nitrogen and phosphorous biotransformation during biological treatment at WWTPs, were also identified in 0.2% and 0.3% of the taxonomic directed reads of the metagenome analyses, respectively. Therefore, the recycling of the retentates to the activated sludge tanks could support the growth of these slow growing beneficial bacteria, on the one hand. On the other hand, facultative pathogenic bacteria were present in similar concentrations and their higher growth rates could support a risk of accumulation of unwanted bacteria. They made up together 0.8% of the complete bacterial population, with *E*. *coli* comprising 0.4%, *A*. *baumannii* 0.3%, and enterococci 0.1%, respectively. Other clinically relevant bacteria were also detected with 0.4% of reads mapping to *P*. *aeruginosa* and 0.01% of reads mapping to *Staphylococcus aureus*. An overview of the composition of the species levels that made up more than 1% of the complete population is listed in SI Fig. 1.

Recycling of these facultative pathogenic bacteria might risk further accumulation, or even proliferation, within the biological treatment tanks. In this case an inactivation stage may be advantageous. It could be shown that the retentate harbours an elevated risk of promoting horizontal gene transfer potential through the high abundances of transposases within the bacterial community. As a consequence, recycling of these retentates back to activated sludge tanks might support horizontal gene transfer and the evolution of antibiotic resistances in bacterial populations. Therefore, a deeper understanding of the behaviour of these different kinds of bacteria after recycling is absolutely necessary for future technical operations at WWTPs.

## Conclusion

**Figure 6:**
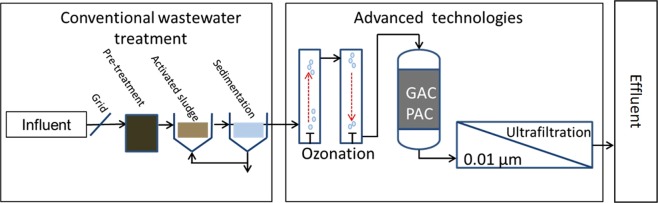
Proposed technical operation at WWTPs for a sufficient elimination of antibiotic resistant bacteria contamination. Conventional wastewater treatment is followed by an ozonisation for micropollutant transformation and first reduction of biological contaminants. An adsorptive treatment process with activated charcoal removes or mineralise possible by-products of the ozonation process by adsorption or biotransformation. Finally, an ultrafiltration is suggested to remove facultative pathogenic bacteria and bacteria carrying antibiotic resistance genes.

The present study is demonstrating that technical measurements are already available to effectively reduce or eliminate antibiotic resistant genes and facultative pathogenic bacteria from municipal wastewaters at treatment plants. Both ultrafiltration and ozonation technologies demonstrated different removal efficiencies, but also distinct benefits and disadvantages. Ultrafiltration, as a physical technology, removed an average of 5 log units of both facultative pathogenic bacteria and ARGs, while ozonation adjusted to micro-pollutant treatment removed about 2 log units on average. In contrast to ultrafiltration, ozonation is known to effectively reduce micro-pollutants like antibiotic drugs or biocides by oxidative transformation, which might be helpful in reducing selective pressure on bacteria. Hence, future studies should include combined technologies like ozonation, with subsequent adsorptive filtration (charcoal or sand filtration), and membrane-based ultrafiltration to prevent environmental contamination, especially with antibiotic resistant, clinically relevant bacteria^26^. Such a proposed combination of oxidative, adsorptive, and membrane-based technology is illustrated in Fig. 6. Recently, it was calculated that about 2.66 × 10^13^ cell equivalents per day (i.e. antibiotic resistance genes and facultative pathogenic bacteria) reached the receiving body after conventional treatment. This discharge was significantly reduced to 6.5 × 10^8^ cell equivalents per day of facultative pathogenic bacteria, including antibiotic resistance genes, when an ultrafiltration is performed. Finally, both technologies are valuable steps to interrupt dissemination of antibiotic resistant bacteria to the environment, as recommended by the WHO and UN assembly. It has to be pointed out that the present data of abundances and reduction efficiencies were obtained from only one specific treatment plant with its particular catchment area. It is difficult to introduce general kind of threshold values for wastewater conditioning processes, e.g. due to individual microbial growth dynamics and gene exchange capacities in receiving waters. Nevertheless, it could be demonstrated that both technologies, ozonation as well as ultrafiltration, were able reduce especially the most clinically relevant, but almost rarely detected ARGs (e.g. *mcr*-*1*, *bla*NDM-*1*, *vanA*, and *bla*VIM) and facultative pathogenic bacteria below the detection limit. In case of higher abundances combinatory efforts are recommended to reduce the antibiotic resistance determinants in a sufficient degree. In regard to technical and economic feasibility, an overall reduction of 4–5 log units (min. 99,99%) including ARGs and facultative pathogenic bacteria in higher abundances through the complete treatment plant could be a reasonable reference value.

## Supplementary information

Dissemination prevention of antibiotic resistant and facultative pathogenic bacteria by ultrafiltration and ozone treatment at an urban wastewater treatment plant

## Data Availability

Metagenomic raw data can be found at the NCBI database with the BioProject Accession number PRJNA524456. The data underlying Figs 2 and 3 are listed in SI Tables 6–9 and concrete p-values indicated in Figs 2 and 3 are displayed in the SI Table 10. The authors declare that the data supporting the findings of this study are available within the paper and its supplementary information files.
