# Supplementary material for: Dissemination prevention of antibiotic resistant and facultative pathogenic bacteria by ultrafiltration and ozone treatment at an urban wastewater treatment plant
Source: Sci Rep. 2019 Sep 6;9:12843. doi: 10.1038/s41598-019-49263-1 (PMC6731226; doi:10.1038/s41598-019-49263-1)
Supplement: Supplementary file 1 — Dissemination prevention of antibiotic resistant and facultative pathogenic bacteria by ultrafiltration and ozone treatment at an urban wastewater treatment plant [file 41598_2019_49263_MOESM1_ESM.docx]

# Dissemination prevention of antibiotic resistant and facultative pathogenic bacteria by ultrafiltration and ozone treatment at an urban wastewater treatment plant

N. Hembach^1^, J. Alexander^1^, C. Hiller^2^, Arne Wieland^3^, T. Schwartz^1*^

SI Figure 1: Population diversity of the retentate of UF1 and UF2.

SI table 1: List of all used primers. Containing the Primer sequences, Fragment size, equation obtained by calibration, efficiency and correlation coefficient of the used calibration curve, used control strain, gene location, and references for primer sequences.

|  | Primer sequences | Calibration equation | Fragment size | Efficiency | R² | control strain | Gene location | reference |
| --- | --- | --- | --- | --- | --- | --- | --- | --- |
|  | Facultative pathogenic bacteria | | | | | | | |
| 16S rRNA | Fwd: TCCTACGGGAGGCAGCAGT  Rev: ATTACCGCGGCTGCTGG | F(x)=-3.406x+36.360 | 195 bp | 96.6% | 0.997 | *E. coli* pNORM | plasmid | Rocha et al. 2018 |
| *E. coli* | Fwd: GCATCGTGACCACCTTGA)  Rev: CAGCGTGGTGGCAAAA) | F(x)= -3.361x+35.797 | 59 bp | 98.4% | 0.994 | *E. coli*  DSM1103 | genome | Clifford et al. 2012 |
| *A. baumannii* | Fwd: GTTGTGGCTTTAGGTTTATTATACG  Rev: AAGTTACTCGACGCAATTCG | F(x)= -3.380x+35.679 | 94 bp | 97.6% | 1.000 | *A. baumannii*  DSM30007 | genome | Clifford et al. 2012 |
| Enterococci  (23S rDNA) | Fwd: AGAAATTCCAAACGAACTTG  Rev: CAGTGCTCTACCTCCATCATT | F(x)= -3.585+35.283 | 93 bp | 90.1% | 1.000 | *E. faecium*  DSM20477 | genome | Volkmann et al. 2004 |
|  | Antibiotic resistance genes | | | | | | | |
| *bla*TEM | Fwd: TTCCTGTTTTTGCTCACCCAG  Rev: CTCAAGGATCTTACCGCTGTTG | F(x)=-3.303x+38.559 | 112 bp | 100.8% | 0.999 | *E. coli* pNORM | plasmid | Rocha et al. 2018 |
| *tetM* | Fwd: GGTTTCTCTTGGATACTTAAATCAATC  Rev: CCAACCATAAATCCTTGTTCRC | F(x)=-3.424x+38.747 | 88 bp | 95.9% | 0.998 | *E. coli*  DH5α | plasmid | Peak et al. 2007 |
| *sul1* | Fwd: CGCACCGGAAACATCGCTGCAC  Rev: TGAAGTTCCGCCGCAAGGCTCG | F(x)=-3.387x+39.802 | 161 bp | 97.6% | 0.999 | *E. coli* pNORM | plasmid | Rocha et al. 2018 |
| *intl1* | Fwd: GCCTTGATGTTACCCGAGAG  Rev: GATCGGTCGAATGCGTGT | F(x)= -3.472x+34.72 | 196 bp | 94.1% | 1.000 | *E. coli* pNORM | plasmid | Rocha et al. 2018 |
| *CTX-M* | Fwd: CGCTTTGCGATGTGCAG  Rev: ACCGCGATATCGTTGGT | F(x)= -3,504x+34,255 | 551 bp | 92,9% | 1.000 | *E. coli* pNORM | plasmid | Rocha et al. 2018  Paterson et al 2003 |
| *CTX-M-32* | Fwd: CGTCACGCTGTTGTTAGGAA  Rev: CGCTCATCAGCACGATAAAG | F(x)= -3,517x+37,80 | 155 bp | 92,5% | 1.000 | *E. coli* pNORM | plasmid | Rocha et al. 2018 |
| *bla*OXA-48 | Fwd: TGTTTTTGGTGGCATCGAT)  Rev: GTAAMRATGCTTGGTTCGC | F(x)= -3,540x+36,913 | 177 bp | 91,6% | 0,998 | *Klebsiella pneumoniae*  TGH isolate 2* | plasmid | Monteiro et al. 2012 |
| *bla*VIM | FWD: GAGATTCCCACGCACTCTCTAGA)  Rev: AATGCGCAGCACCAGGATAG  Probe: ACGCAGTGCGCTTCGGTCCAGT | F(x)= -3,829x+40,868 | 61 bp | 82,5% | 0,999 | *Pseudomonas aeruginosa*  PA49 isolate** | unknown | Van der Zee et al., 2014 |
| *CMY-2* | FWD: CGTTAATCGCACCATCACC  REV: CGTCTTACTAACCGATCCTAGC | F(x)=-3,591x+34,026 | 172 bp | 89,9% | 0,998 | *Klebsiella pneumoniae*  NRZ-01013 | unknown | Kurpiel and Hanson 2011 |
| *vanA* | FWD: TCTGCAATAGAGATAGCCGC  Rev: GGAGTAGCTATCCCAGCATT | F(x)=-3,541x+33,078 | 376 bp | 91,6% | 1,000 | *Enterococcus faecium*  B7641 vanA | unknown | Klein et al. 1998 |
| *mcr-1* | Fwd: GGGCCTGCGTATTTTAAGCG  Rev: CATAGGCATTGCTGTGCGTC | F(x)=-3,386x+35,349 | 183 bp | 97,4% | 0,999 | *E. coli*  NRZ-14408 | plasmid | Hembach et al. 2017 |
| *bla*NDM | Fwd: TTGGCCTTGCTGTCCTTG  Rev: ACACCAGTGACAATATCACCG | F(x)=-3,293x+35,877 | 82 bp | 101,2% | 0,999 | *Klebsiella pneumoniae*  ATCC BAA-2146 | plasmid | Monteiro et al., 2012 |

*National Reference Center for Gram-negative pathogens, Ruhr University in D-44801 Bochum, Germany

** *Pseudomonas aeruginosa*, PA49: multi-resistant isolate from hospital wastewater (see: Berditsch, M., Jäger, T., Strempel, N., Schwartz, T., Overhage, J., & Ulrich, A. S. (2015). Synergistic effect of membrane-active peptides polymyxin B and gramicidin S on multidrug-resistant strains and biofilms of *Pseudomonas* *aeruginosa*. Antimicrobial Agents and Chemotherapy, 59(9), 5288-5296.)

SI table 2: Detection limits of each detection system in cell equivalents/ PCR reaction.

|  | Targets | cell equivalents/ PCR reaction |
| --- | --- | --- |
|  | *Eubacteria* | 10600 |
| facultative pathogenic bacteria | *E. coli* | 4,01 |
|  | *A. baumann*ii | 31 |
|  | Enterococci (23S rDNA) | 64,8 |
| Antibiotic resistance genes | *bla*TEM | 80,1 |
|  | *tetM* | 3,69 |
|  | *sul1* | 80,18 |
|  | *intl1* | 126 |
|  | *CTX-M* | 93,1 |
|  | *CTX-M-32* | 235 |
|  | *bla*OXA-48 | 92,3 |

SI table 3: Cell equivalents in 100 mL of facultative pathogenic bacteria and ARGs after biological treatment and a subsequent filtration with both UF units at different Flux rates (12.5, 50, 90 L/m²h). Due to operative reasons each parameter could only get tested two times (n=2). LOD: Limit of detection.

|  | | UF1 | | | | UF2 | | |
| --- | --- | --- | --- | --- | --- | --- | --- | --- |
|  |  | Influent | Flux 12,5 L/m²h | Flux 50 L/m²h | Flux 90 L/m²h | Influent | Flux 12,5 L/m²h | Flux 50 L/m²h |
|  | *Eubacteria* | 3.46E+06 | 4.19E+06 | 2.65E+05 | 5.81E+04 | 9.95E+08 | 2.50E+04 | 3.05E+04 |
| Facultative pathogenic bacteria | *E. coli* | 2.79E+02 | 4.85E+00 | <LOD | <LOD | 1.58E+04 | <LOD | 1.66E+00 |
|  | *A. baumannii* | 3.09E+01 | 1.06E+00 | <LOD | <LOD | 4.03E+02 | <LOD | >LOD |
|  | Enterococci | 1.49E+02 | 1.21E+01 | 3.11E-01 | <LOD | 1.79E+04 | 9.93E-01 | 2.48E+00 |
| ARGs | *bla*TEM | 3.09E+03 | 7.08E+01 | 1.13E+01 | <LOD | 2.03E+05 | 2.09E+02 | 1.01E+02 |
|  | *tetM* | 5.34E+02 | <LOD | <LOD | <LOD | 7.30E+04 | <LOD | 1.08E+01 |
|  | *sul1* | 1.72E+05 | 2.53E+03 | 1.42E+02 | 2.48E+01 | 1.76E+05 | <LOD | 2.18E+03 |
|  | *intl1* | 1.65E+04 | 1.02E+04 | 4.02E+02 | <LOD | 2.45E+04 | 3.59E+01 | 2.73E+02 |
|  | *CTX-M* | <LOD | <LOD | <LOD | <LOD | 4.80E+05 | <LOD | <LOD |
|  | *CTX-M-32* | <LOD | <LOD | <LOD | <LOD | 1.03E+06 | <LOD | <LOD |
|  | *bla*OXA-48 | 6.56E+01 | <LOD | <LOD | <LOD | 1.83E+04 | <LOD | 1.18E+00 |

SI table 4: Cell equivalents in 100 mL of facultative pathogenic bacteria and antibiotic resistance genes after biological treatment (Influent) and in the retentate of both ultrafiltration units.

|  | influent UF1 | retentate UF1 | influent UF2 | retentate UF2 |
| --- | --- | --- | --- | --- |
| *Eubacteria* | 3,46E+06 | 2,53E+08 | 9,95E+08 | 6,80E+08 |
| *E. coli* | 2,79E+02 | 1,25E+04 | 1,58E+04 | 1,39E+05 |
| *A. baumannii* | 3,09E+01 | 3,30E+02 | 4,03E+02 | 2,01E+03 |
| Enterococci | 1,49E+02 | 1,40E+04 | 1,79E+04 | 8,32E+04 |
|  |  |  |  |  |
| *bla*TEM | 3,09E+03 | 1,20E+05 | 2,03E+05 | 1,07E+06 |
| *ermB* | 1,18E+03 | 7,15E+04 | 9,96E+04 | 7,60E+05 |
| *tetM* | 5,34E+02 | 2,76E+04 | 7,30E+04 | 3,18E+05 |
| *bla*OXA-48 | 6,56E+01 | 2,95E+03 | 1,83E+04 | 3,19E+04 |
| *sul1* | 1,72E+05 | 2,41E+06 | 1,76E+05 | 1,38E+07 |
| *intl1* | 1,65E+04 | 7,49E+05 | 2,45E+04 | 4,94E+06 |
| *CTX-M* | <LOD | 1,08E+03 | 4,80E+05 | 2,23E+04 |
| *CTX-M-32* | <LOD | 9,17E+03 | 1,03E+06 | 1,14E+05 |

SI table 5: List of all found resistance genes in the retentate during metagenomic sequencing.

| Methicillin resistance | Erythromycin resistance | Tetracyline resistances | Vancomycine resistance | | Imipenem resistance | Beta- lactamases | Metallo beta-lactamases |
| --- | --- | --- | --- | --- | --- | --- | --- |
| *mecA*  *mecR1* | *ermB*  *ermBP*  *ermC*  *ermF*  *ermG*  *ermGN*  *ermX* | *tetA*  *tetB*  *tetD*  *tetG*  *tetH*  *tetL*  *tetM*  *tetO*  *tetQ*  *tetR*  *tetS*  *tetT*  *tetW*  *tetX2*  *tetY* | *VanA*  *VanA2*  *VanB*  *VanD*  *VanD5*  *VanE*  *VanG*  *VanH*  *VanH*  *VanHB*  *VanR*  *VanRB*  *VanRD*  *VanRE*  *VanS*  *VanS* | *VanSB*  *VanSC*  *VanSD*  *VanSE*  *VanTG*  *VanU*  *VanW*  *VanWB*  *VanX*  *VanXB*  *VanXB*  *VanY*  *Vanyb*  *VanYG*  *VanZ* | *bla*Vim1  *bla*Vim2  *bla*Vim3  *bla*Vim4  *bla*Vim5  *bla*Vim8  *bla*Vim9  *bla*Vim10  *bla*Vim11  *bla*Vim12  *bla*Vim18 | *bla*OXA-2  *blaOXA-9*  *blaOXA-46*  *bla*OXA-53  *bla*OXA-28  *bla*OXA-10  *bla*OXA-1  *bla*OXA-47  *CTX-M-7*  *CTX-M-33*  *CTX-3*  *CTX-M-9*  *CTX-M-2*  *CTX-M-30*  *CTX-M-1*  *CTX-M-37*  *CTX-M-15*  *CTX-M-26*  *bla*TEM  *Various others* | *bla*KPC  *bla*NDM  Various others |

SI Table 6: Median, quartiles, minimal and maximal abundances of facultative pathogenic bacteria and ARGs in the effluent of the conventional treatment in cell equivalents/ 100 mL. n= 4, LOD: Limit of detection.

| Boil. treatment | 16S rRNA | *E. coli* | *A. baumannii* | Enterococci | *bla*TEM | *tetM* | *sul-1* | *intl1* | *CTX-M* | *CTX-M-32* | *bla*OXA-48 |
| --- | --- | --- | --- | --- | --- | --- | --- | --- | --- | --- | --- |
| minimum | 4.32E+08 | 1.75E+02 | <LOD | 5.20E+01 | 8.27E+04 | 1.57E+04 | 3.95E+06 | 2.26E+04 | 5.04E+02 | 1.17E+04 | 1.38E+03 |
| 0.25 quantile | 5.30E+08 | 8.43E+03 | 3.26E+02 | 3.02E+03 | 2.98E+05 | 4.26E+04 | 7.75E+06 | 1.69E+06 | 1.33E+03 | 1.39E+04 | 1.60E+03 |
| median | 6.28E+08 | 2.01E+04 | 1.63E+03 | 7.83E+03 | 4.72E+05 | 6.31E+04 | 1.25E+07 | 2.00E+06 | 2.10E+03 | 1.52E+04 | 5.81E+03 |
| 0.75 quantile | 1.16E+09 | 3.40E+04 | 2.79E+03 | 1.81E+04 | 1.58E+06 | 1.53E+05 | 1.84E+07 | 3.92E+06 | 3.55E+03 | 1.83E+04 | 4.63E+04 |
| maximum | 1.69E+09 | 1.54E+05 | 3.04E+03 | 4.62E+04 | 9.28E+06 | 3.89E+05 | 2.57E+07 | 8.32E+06 | 6.44E+03 | 6.76E+04 | 1.55E+05 |

SI Table 7: Median. quartiles. minimal and maximal abundances of facultative pathogenic bacteria and ARGs in the effluent of the ozone facility in cell equivalents/ 100 mL. n= 4. LOD: Limit of detection.

| ozone | 16S rRNA | *E. coli* | *A. baumannii* | Enterococci | *bla*TEM | *tetM* | *sul-1* | *intl1* | *CTX-M* | *CTX-M-32* | *bla*OXA-48 |
| --- | --- | --- | --- | --- | --- | --- | --- | --- | --- | --- | --- |
| minimum | 3.06E+06 | 4.66E+01 | <LOD | 8.62E+00 | 4.90E+03 | 1.97E+02 | <LOD | 6.39E+03 | <LOD | 3.03E+02 | 0.00E+00 |
| 0.25 quantile | 3.46E+06 | 7.47E+01 | <LOD | 2.56E+01 | 6.93E+03 | 2.10E+02 | 1.30E+04 | 1.89E+04 | <LOD | 4.67E+02 | 0.00E+00 |
| median | 3.75E+06 | 1.13E+02 | <LOD | 8.18E+01 | 1.45E+04 | 2.18E+02 | 3.92E+04 | 2.44E+04 | <LOD | 2.77E+03 | 0.00E+00 |
| 0.75 quantile | 1.72E+07 | 5.90E+03 | 1.86E+00 | 2.06E+02 | 2.46E+04 | 3.64E+02 | 7.42E+04 | 2.93E+04 | <LOD | 5.86E+03 | 0.00E+00 |
| maximum | 5.71E+07 | 2.32E+04 | 7.43E+00 | 4.25E+02 | 3.41E+04 | 7.87E+02 | 1.13E+05 | 4.00E+04 | <LOD | 8.35E+03 | 0.00E+00 |

SI Table 8: Median. quartiles. minimal and maximal abundances of facultative pathogenic bacteria and ARGs in UF1 effluent in cell equivalents/ 100 mL. n= 4. LOD: Limit of detection.

| UF1 | 16S rRNA | *E. coli* | *A. baumannii* | Enterococci | *bla*TEM | *tetM* | *sul-1* | *intl1* | *CTX-M* | *CTX-M-32* | *bla*OXA-48 |
| --- | --- | --- | --- | --- | --- | --- | --- | --- | --- | --- | --- |
| minimum | <LOD | <LOD | <LOD | <LOD | <LOD | <LOD | <LOD | <LOD | <LOD | <LOD | <LOD |
| 0.25 quantile | 4.54E+02 | <LOD | <LOD | <LOD | 1.09E+01 | <LOD | <LOD | 5.55E-01 | <LOD | <LOD | <LOD |
| median | 1.35E+03 | 5.52E-01 | <LOD | 7.03E-02 | 2.18E+01 | <LOD | <LOD | 1.11E+00 | <LOD | <LOD | <LOD |
| 0.75 quantile | 3.17E+03 | 1.38E+00 | <LOD | 3.49E-01 | 3.28E+01 | <LOD | <LOD | 1.66E+00 | <LOD | <LOD | <LOD |
| maximum | 6.38E+03 | 2.21E+00 | <LOD | 9.75E-01 | 4.37E+01 | <LOD | <LOD | 2.22E+00 | <LOD | <LOD | <LOD |

SI Table 9: Median. quartiles. minimal and maximal abundances of facultative pathogenic bacteria and ARGs in UF2 effluent in cell equivalents/ 100 mL. n= 4. LOD: Limit of detection.

| UF2 | 16S rRNA | *E. coli* | *A. baumannii* | Enterococci | *bla*TEM | *tetM* | *sul-1* | *intl1* | *CTX-M* | *CTX-M-32* | *bla*OXA-48 |
| --- | --- | --- | --- | --- | --- | --- | --- | --- | --- | --- | --- |
| minimum | 3.23E+03 | <LOD | <LOD | <LOD | 0.00E+00 | <LOD | <LOD | <LOD | <LOD | <LOD | <LOD |
| 0.25 quantile | 1.14E+04 | <LOD | <LOD | <LOD | 1.41E+01 | <LOD | 2.87E+01 | 2.82E+01 | <LOD | <LOD | <LOD |
| median | 2.19E+04 | 6.13E-01 | <LOD | 2.62E-01 | 2.81E+01 | <LOD | 5.75E+01 | 4.35E+01 | <LOD | <LOD | <LOD |
| 0.75 quantile | 8.36E+04 | 2.15E+00 | <LOD | 1.39E+00 | 2.95E+01 | <LOD | 7.95E+01 | 6.14E+01 | <LOD | <LOD | <LOD |
| maximum | 2.46E+05 | 4.91E+00 | <LOD | 4.00E+00 | 3.10E+01 | <LOD | 1.01E+02 | 9.73E+01 | <LOD | <LOD | <LOD |

SI Table 10: p-values resulting from one tailed Mann-Whitney-U test for a significant reduction outgoing from boil. treatment to the referred treatment step.

| p-value | 16s rRNA | *E. coli* | *A. baumannii* | Enterococci | *bla*TEM | *tetM* | *sul-1* | *intl1* | *CTX-M* | *CTX-M-32* | *bla*OXA-48 |
| --- | --- | --- | --- | --- | --- | --- | --- | --- | --- | --- | --- |
| ozone | 0.02591 | 0.9669 | 0.03666 | 0.0331 | 0.00998 | 0.01519 | 0.01519 | 0.05567 | 0.01054 | 0.00998 | 0.01054 |
| UF1 | 0.02591 | 0.00973 | 0.02209 | 0.00973 | 0.04068 | 0.01054 | 0.01054 | 0.04068 | 0.01054 | 0.00754 | 0.01054 |
| UF2 | 0.02591 | 0.00973 | 0.02209 | 0.00973 | 0.01744 | 0.01054 | 0.02591 | 0.00998 | 0.01054 | 0.00754 | 0.01054 |
